# Supplementary material for: Polyketide synthase gene clusters in the ericoid mycorrhizal fungus Oidiodendron maius: genome-wide prediction, expression profiling, and functional characterization of knockout mutants
Source: Front Plant Sci. 2026 Sep 16;17:1924430. doi: 10.3389/fpls.2026.1924430 (PMC13623903; doi:10.3389/fpls.2026.1924430)
Supplement: Supplementary file 1 [file SupplementaryFile1.zip › Supplementary Materials/Supplementary Folder 1.zip!/Oidma1_AssemblyScaffolds_Repeatmasked/knownclusterblast/cluster57/jgi.p_Oidma1_107057_mibig_hits.html]

| MIBiG Protein | Description | MIBiG Cluster | MiBiG Product | % ID | % Coverage | BLAST Score | E-value |
| --- | --- | --- | --- | --- | --- | --- | --- |
| CBF85175.1 | conserved\_hypothetical\_protein | BGC0000673 | Ent-pimara-8(14),15-diene\_biosynthetic\_gene\_cluster | 40 | 35.0746268657 | 171 | 4.2e-42 |
| AGQ21998.1 | hypothetical\_protein | BGC0000121 | Pestheic\_acid\_biosynthetic\_gene\_cluster | 40 | 30.447761194 | 138 | 3.9e-32 |
